# Differential Cytotoxicity Responses by Dog and Rat Hepatocytes to Phospholipogenic Treatments

**DOI:** 10.1155/2013/956404

**Published:** 2013-03-13

**Authors:** James K. Morelli, Paul J. Ciaccio

**Affiliations:** ^1^Disposition, Safety and Animal Research, Sanofi, 5 The Mountain Road, Framingham, MA 01701, USA; ^2^Global Safety Assessment, AstraZeneca Pharmaceuticals, B2.86, 35 Gatehouse Drive, Waltham, MA 02451, USA

## Abstract

Dog and rat hepatocytes were treated with phospholipogenics to identify the more sensitive species and to determine whether lysosomal or mitochondrial changes were the primary cause of cytotoxicity. Endpoints included cell death, lysosome membrane integrity, mitochondrial membrane polarization, and fluorescent phospholipid (NBD-PE). Dog cells exhibited lower survival IC_50_ values than did rat cells with all phospholipogenic treatments and exhibited a lower capacity to accumulate NBD-PE in 4 of 5 phospholipogenic test conditions. The lysosomal modulator Bafilomycin A1 (Baf) rescued dog cells from cytotoxicity caused by 3 phospholipogenic 5HT1_b_ antagonists and hydroxychloroquine, but not fluoxetine, and rescued rat cells from hydroxychloroquine and NMTMB, a 5HT1_b_ antagonist. Following NMTMB treatment, rat mitochondrial membrane hyperpolarization was observed at modestly cytotoxic concentrations and depolarization at the highest concentration. At the highest test concentration, lysosomal loss of acridine orange occurred by 30 min, mitochondrial polarity changes by 1 hr, and NBD-PE accumulation by 2 hr, respectively. Baf shifted mitochondrial polarity from a depolarized state to a hyperpolarized state. These data demonstrate that (a) dog hepatocytes were generally less capable of mounting an adaptive, protective phospholipidotic response than rat hepatocytes, (b) effects on mitochondria and survival were preventable by lysosomal protection, and (c) destabilizing changes in both organelles are involved causally in cytotoxicity.

## 1. Introduction

Cationic amphiphilic drugs have chemical structures composed of hydrophilic and lipophilic regions, combined with a cationic amine group. Many cationic amphiphilic drugs are phospholipogenic (here defined as a compound that causes phospholipidosis (PLD)). A phospholipidotic response is characterized by the accumulation of phospholipids, lamellar bodies, and drug in cellular lysosomes and results from inhibited lysosomal phospholipase activity, either by binding of drug to phospholipids or by direct inhibition of phospholipases, and through alkalization of the normally acidic lysosomal mileu [1]. Lysosomotropic properties and accumulation potential of phospholipogenics are underscored by studies employing lysosomal modulators. The ionophore monensin, ammonium chloride (which causes direct lysosomal alkalinization), and the specific vacuolar sodium-potassium proton pump inhibitor Bafilomycin A1 (Baf) have been shown to abolish the pH gradient from cytoplasm (near neutral) to lysosome (low internal pH) and preclude accumulation of phospholipogenics in the lysosome and/or rescue cells *in vitro* from such treatments [2–6].

PLD can occur in multiple tissue types, often reflecting benign, adaptive and reversible changes in affected tissues without functional consequence [7, 8], but is sometimes associated with frank tissue degeneration including that for liver and neuronal tissue [9–14]. Drug-induced liver injury is an important source of attrition for pharmaceutical compounds. It does not necessarily result in abandonment of compound development, as toxicity responses across a dosing interval do not always translate across species, and the therapeutic windows may be sufficient. While cause and effect between PLD and degenerative changes has not been clearly established, mechanisms of degenerative concurrent injury caused by phospholipogenics and species response differences should be elucidated where possible to support compound progression into the clinic.

Select phospholipogenics cause liver toxicity in preclinical species such as rats [9–11, 13, 14] and dogs [14]. For example, at comparable plasma exposures in 1-month toxicology studies, a weak phospholipogenic 5HT1_b_ antagonist compound A caused liver PLD in both dog and rat liver but only dog had concomitant centrilobular necrosis (unpublished observations). These findings led us to inquire whether the dog is a more sensitive species generally to phospholipogenic drug administration and whether the phospholipidotic response is a primary cause of cytotoxicity.

We report on *in vitro* investigations showing that, against a panel of such treatments and toxicity endpoints, dog hepatocytes profiled differently than rat hepatocytes as they were more sensitive to the phospholipogenics tested (including compound A) and generally exhibited a diminished capacity to mount protective phospholipidotic responses. Investigations with another 5HT1_b_ antagonist, N-[(2R)-5-methyl-8-(4-methylpiperazin-1-yl)tetralin-2-yl]-4-morpholino-benzamide (NMTMB), suggested that both lysosome and mitochondria organelles are involved in early steps of cell stress. Baf cotreatment rescued hepatocytes from phospholipogenic-mediated cytotoxicity, suggesting that a lysosomal mechanism of cytotoxicity may be primary. Mitochondrial mechanisms of rat cell cytotoxicity, such as mitochondrial membrane depolarization, occurred coordinately with lysosomal membrane destabilization and earlier than detectable phospholipidotic responses.

## 2. Materials and Methods

### 2.1. Cell Culture

Primary rat hepatocytes were freshly isolated from Han Wistar rats at AstraZeneca, using the collagenase perfusion method (Selgen, PO, 1976). Primary dog hepatocytes were isolated at AstraZeneca, or purchased from CellzDirect Corp. (Durham, NC), which also uses a collagenase perfusion method. Cells were centrifuged and resuspended in hepatocyte culture media, consisting of Williams E basal media (Sigma-Aldrich, Inc., St. Louis, MO) 5% Hyclone fetal bovine serum (ThermoFisher Scientific Inc., Rockford, IL), 15 mM HEPES (Mediatech Inc., Manassas, VA), 100 units/mL penicillin/streptomycin, 200 *μ*M l-glutamine, 1X ITS (insulin, transferrin, selenium solution from Mediatech Inc.), and 30 nM dexamethasone (Sigma-Aldrich). Cells were suspended at 2.5 × 10^5^ cell/mL and added to BD Biocoat collagen I coated microtiter plates at 100 *μ*L/well, for a final count of 25,000 cells/well. Selection criterion for use of initial isolates was 85% minimal viability. Cells were incubated at 37°C in a CO_2_ incubator for 4 h to allow for cell attachment. Media was aspirated and replaced with hepatocyte culture media without fetal bovine serum and supplemented with 150 *μ*g/mL Matrigel collagen matrix (BD Inc.). Cells were incubated in a CO_2_ incubator for 24 h, the media was replaced with hepatocyte culture media, and the cells were incubated for an additional 24 h. Compounds were solubilized in DMSO, water, or ethanol to 1000X stock and then diluted to 1X in media. All conditions contained equal concentrations of vehicle. Cells were incubated with drug for the durations indicated in figure or table legends.

### 2.2. Compounds

**Figure 1:**
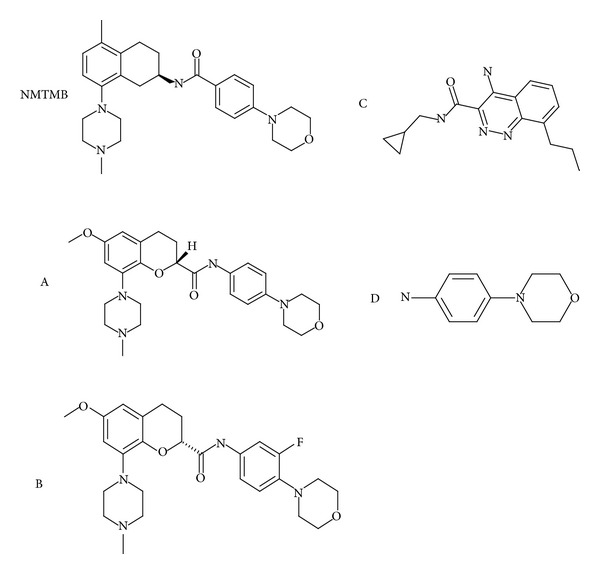
Chemical structures of select compounds used in this study.

Figure 1 shows the chemical structures of AstraZeneca Pharmaceuticals (AZ) 5HT-1_b_ antagonist compounds NMTMB, A, and B. It also shows negative control compounds C and D. All AZ compounds employed in this study were prepared by the AstraZeneca CNS Chemistry Department (Wilmington, DE) and were of >95% chemical and isomeric purity. 5-HT1_b_ receptor antagonists and synthetic steps are further described by Stenfors et al. [15] and Bernstein et al. [16]. NMTMB and compound A are confirmed multiorgan *in vivo* phospholipogenics as they were tested preclinically in both rat and dog models (data not shown). Compound B was tested independently in a splenocyte model *in vitro* [17] and was found to be phospholipogenic. Three compounds (Diclofenac, C, and D), which lack cationic amphiphilic properties, were also tested in this model and were found to be negative. They were therefore used as negative controls in this study. Compound D is the right hand side aniline of A. Diclofenac (obtained from Sigma-Aldrich) is not phospholipogenic *in vivo*. Additional positive controls, obtained from Sigma-Aldrich, were fluoxetine and hydroxychloroquine and are known to cause PLD (see summary reviews [1, 7, 9]). The vacuolar ATPase proton pump inhibitor Bafilomycin A1 (Sigma-Aldrich) has been shown to alkalinize lysosomes and prevent lysosomal membrane permeability and intrinsic apoptosis [18, 19]. PADK (Z-Phe-Ala-diazomethylketone) (Bachem Bioscience, Inc. King of Prussia, PA) is known to enhance intralysosomal cathepsin activity [20].

The Baf concentration selection for these experiments was based upon several observations. At 30 nM Baf exhibited no effects on control (ATP levels, not shown) or survival. One hundred nM Baf caused 2-fold increases in dog and rat hepatocyte control NBD-PE fluorescent staining, but minimal changes at 30 nM. One hundred nM Baf but not 30 nM caused vacuolation of dog hepatocytes. Finally, 100 nM but not 30 nM Baf enhanced cell death in rat hepatocytes when cotreated with hydroxychloroquine and amiodarone treatments. This concentration selection and outcomes differ from that of the work of Boya et al. [18] who employed CHO cells, a cell line which possesses a very different phenotype than primary hepatocytes.

### 2.3. NBD-PE Accumulation Assay

In a modification of the procedure described by Morelli et al. [17], hepatocytes were treated with drugs diluted into culture media containing 12 *μ*g/mL NBD-PE (N-(7-nitrobenz-2-oxa-1,3-diazol-4-yl)-1,2-dihexadecanoyl-*sn*-glycero-3-phosphoethanolamine, triethylammonium salt) for 24 hours. At the end of the treatment period, Sytox orange (100 nM Invitrogen Inc.) was added to cells for 20 minutes prior to fixation. This fluorescent dye stains only cells with compromised cell membranes thereby enabling their exclusion from NBD-PE cell quantitation by the Cellomics ArrayScan instrument. Media were aspirated, cells were washed with 1X PBS and fixed by the addition of 100 *μ*L PBS containing 3.7% formaldehyde, and 1 *μ*g/mL Hoechst dye. Fixation was allowed to proceed for 30 min at RT, and cells were washed three times with 1X PBS, and sealed for imaging. Labeled cells were analyzed using the Cellomics Arrayscan instrument (Cellomics Inc., Pittsburgh, PA) with version 3.5 equipment and software. The ArrayScan captures fluorescent images in the 3 channels relevant to this assay (Hoechst, TRITC for Sytox staining and FITC for NBD-PE staining) and performs automated image analysis to quantify cell nuclei, the number of dead cells, and NBD-PE dye fluorescent intensity. The analysis algorithms that enable cell versus well-based detection were purchased from Cellomics Inc. and are referred to as Cell Health Profiling (CHP) and Target Activation (TA) algorithms. Given bell-shaped concentration-response curves, accurate EC_50_ (half maximal effective concentration) values were difficult to obtain but were estimated from the left-hand portion of the concentration response curves. For time-course investigations with NMTMB, concentrations were selected to bracket the rat survival IC_50_ value and to approach the NBD-PE EC_50_ (~65 *μ*M) value. NBD-PE peak signal was defined as the maximal response.

### 2.4. Acridine Orange Time Course

Acridine Orange (AO) (Invitrogen Inc.) was purchased at a concentration of 10 mg/mL in water, and diluted to 50 *μ*M final in Williams E media. Cells were preincubated for 1 h in 90 *μ*L complete Williams E media, or complete Williams E media containing 30 nM Bafilomycin A1 for 1 h. Ten *μ*L Acridine Orange was added to wells to reach a final well concentration of 5 *μ*M. Cells were incubated with AO for 10 minutes. The media were then aspirated, the cells rinsed once with PBS, and replaced with fresh media containing compounds of interest, plus or minus Baf. As above, labeled cells were analyzed using the Cellomics Arrayscan instrument (Cellomics Inc., Pittsburgh, PA).

### 2.5. Mitochondrial Membrane Potential

JC-1 fluorescent dye (Invitrogen Inc., Carlsbad, CA) is used to monitor mitochondrial health since it exhibits membrane potential-dependent accumulation in mitochondria reflected as a fluorescence emission shift from green (~529 nm) to red (~590 nm). Mitochondrial depolarization is indicated by a decrease in the red/green fluorescence intensity ratio (hyperpolarization exhibiting the opposite). JC-1 was added to cell cultures at a concentration of 5 *μ*g/mL for 30 minutes to allow for cellular accumulation. Media were then aspirated and replaced with fresh media containing drugs of interest without JC-1. Fluorescence was detected using a spectrofluorometer. The ratio of red to green signals was calculated and converted to % control for graphing and analysis.

## 3. Results

### 3.1. Differential Survival and NBD-PE Accumulation by Rat versus Dog Hepatocytes in Response to Phospholipogenic Compounds

**Figure 2:**
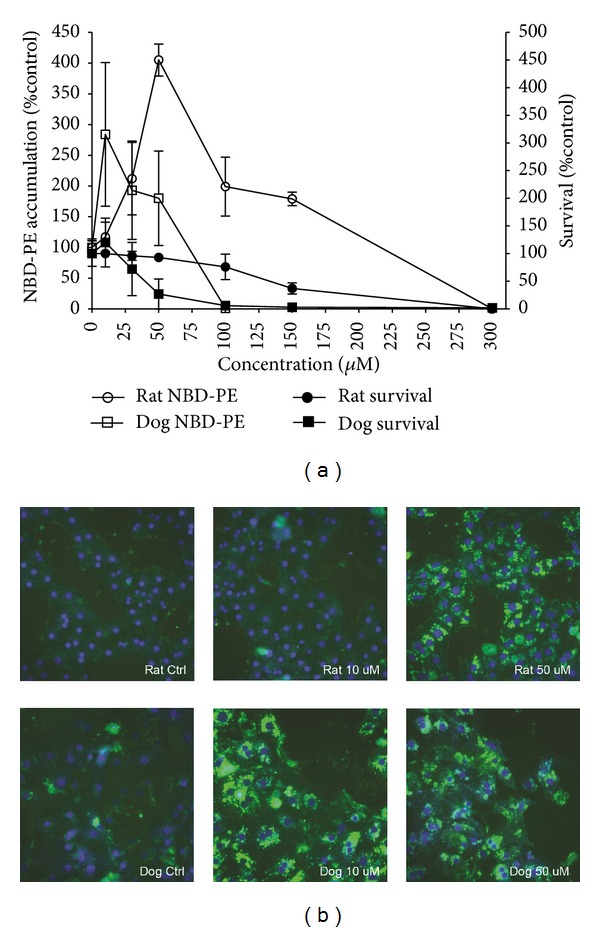
(a) Differential effect of NMTMB on survival and NBD-PE accumulation in dog and rat hepatocytes. Cells were treated with compound in NBD-PE containing media. After 24 h cells were fixed with formaldehyde/Hoechst, and both survival and NBD-PE accumulation were quantified using the Cellomics Arrayscan instrument (as described in Section 2). Expressed as % control ±SD for 3 replicates from single isolations. Dog and rat hepatocytes were isolated for this experiment on the same day. (b) Differential effect of NMTMB on accumulation of NBD-PE in rat and dog hepatocytes. Cells were treated with NMTMB in NBD-PE containing media. After 24 h cells were fixed with formaldehyde/Hoechst and imaged using the Cellomics Arrayscan instrument. Image exposures were identical in all experiments (0.5 sec); background levels of NBD-PE accumulation were similar between dog and rat hepatocytes (approximately 50 mean average intensity units). Some artifactual accumulation of NBD-PE in dog preparations is apparent in the control sample. Dog and rat hepatocytes were isolated for this experiment on the same day.

To determine which species' hepatocytes were more sensitive to phospholipogenic compounds, cells were treated for 24 hr with the potent phospholipogenic NMTMB, and endpoints of cell death and phospholipidotic response were monitored. Compared to rat hepatocytes, dog hepatocytes were more sensitive to treatment with this compound (Figure 2(a)). In this comparative experiment complete cell death occurred in dog hepatocytes at 100 *μ*M, whereas ~80% of rat hepatocytes typically survived at this concentration. Fluorescent phospholipid NBD-PE accumulation in live cells also differed markedly between the 2 species. Dog cells exhibited greater phospholipidotic response potency (illustrated by low test concentration responses that were not observed in rat cell conditions) but with a lower maximal response (% vehicle control).  Figure 2(b) shows representative fluorescent images of NBD-PE containing hepatocytes illustrating these findings visually. At 10 *μ*M NMTMB, NBD-PE accumulation was lower in rat hepatocytes than in dog hepatocytes. At 50 *μ*M, rat cells exhibited significant NBD-PE accumulation (4-fold vehicle control), a majority of the cells were viable and NBD-PE responses were characterized morphologically by a punctuate-granular appearance. In contrast, at this concentration dog cell NBD-PE accumulation was diminishing, fluorescence was both punctuate-granular and diffuse in appearance, and ~70% cells were dead, accounting for the bell-shape of NBD-PE curves in these isolates (illustrated in Figure 2(a)).

**Figure 3:**
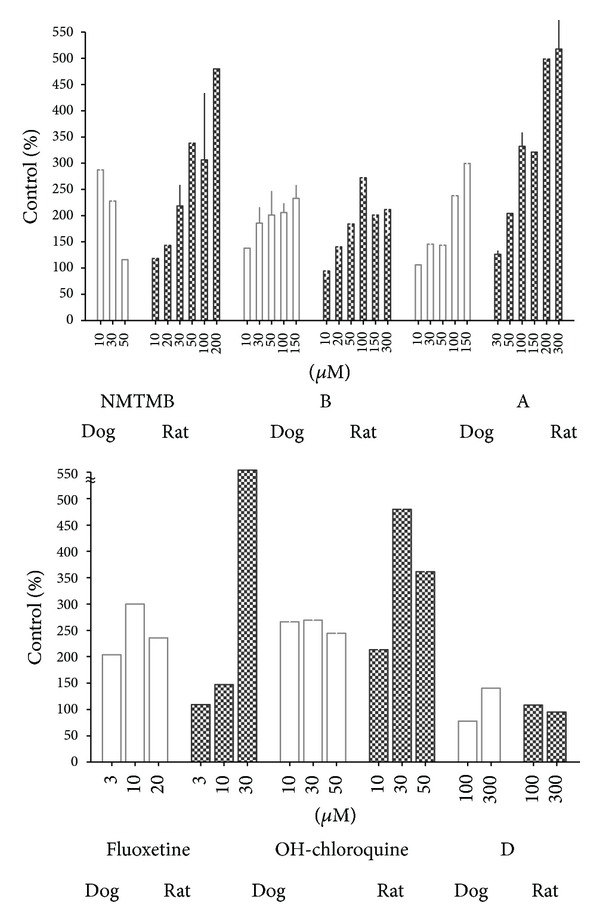
Differential effects of phospholipogenic test articles on accumulation of the fluorescent phospholipid NBD-PE on dog and rat hepatocytes. Cells were treated with known phospholipogenic or nonphospholipogenic compounds in NBD-PE containing media. After 24 h cells were fixed with formaldehyde/hoechst, and NBD-PE accumulation was quantified using the Cellomics Arrayscan instrument. Image exposures were identical in all experiments (0.5 sec); background levels of NBD-PE accumulation were similar between dog and rat hepatocytes (approximately 50 mean average intensity units). Accumulation is expressed as % VC (vehicle control) at listed concentrations. Three replicates for each cell isolate were employed per experiment. Isolations occurred on separate days. The data are represented as an average of individual experiments where *N* = 1-2 (where histograms do not have error bars) or an average of 3 independent isolates ±SEM.

To determine whether this species response differential was general, we tested a larger set of phospholipogenics, alongside a few nonphospholipogenic compounds (negative controls). This larger test set included compound A, a 5HT1_b_ antagonist that caused in vivo multiorgan PLD in 1 month repeat-dose toxicology studies, including liver PLD in both dog and rat, and centrilobular loss in dog liver only (data not shown). Statistical comparisons across species were not applied in this particular instance since the data, while reflecting an average of three well replicates per experiment, only represent in some instances an average of *N* = 1 or 2 individual (isolate) experiments (where histograms in the figure do not have error bars) (averages of 3 independent isolates have ±SEM error bars). Results however across independent experiments exhibit clear trends. Compared to rat hepatocytes, phospholipidotic responses by dog hepatocytes were more potent for 2 of 5 phospholipogenic compounds tested (NMTMB and Fluoxetine, Figure 3), but exhibited a lower capacity to accumulate NBD-PE compared to rat hepatocytes at higher concentrations for these two as well, compound A and hydroxychloroquine. Rat hepatocytes accumulated as much as 5-fold vehicle control levels of NBD-PE (% VC or maximal response), whereas dog hepatocytes never exceeded 3-fold vehicle control levels (Figure 3) (note: background NBD-PE levels detected by the Cellomics Arrayscan were similar between control dog and rat hepatocytes.). Compound A treatment did not yield a less potent NBD-PE response in rat hepatocytes compared to dog, but the maximal response in hepatocytes from this species was on average greater than 1.7-fold that for dog cells. In contrast to that for phospholipogenic treatment conditions, absolute levels of NBD-PE accumulation were minimal and differential effects in phospholipidotic potency or maximal response were not observed with negative control compounds in hepatocytes isolated from these two species (compound D exhibited 140% (dog) and 101% (rat) control activity at 300 *μ*M (*N* = 1, Figure 3); compound C exhibited 121% (dog) and 95% (rat) control activity at 300 *μ*M (*N* = 1, data not shown)).

Like responses to NMTMB, rat hepatocytes were more resistant to cytotoxicity caused by all other phospholipogenic compounds tested, including compound A (Table 1). Rat hepatocyte survival IC_50_ (half maximal inhibitory concentration) values were generally 2- to 3-fold greater than those for dog cells. For some rat treatment conditions, a convincing quantitative difference in survival could not be specifically determined given solubility limitations in DMSO, as concentrations required for IC_50_ (half maximal inhibitory concentration) interpolation would be above the highest concentrations tested (listed in Table 1 as >300 *μ*M or >450 *μ*M). It was thus clear that rat hepatocytes tolerated these compounds better than dog hepatocytes since rat cell survival was minimally affected at 300 or 450 *μ*M. Response differentials for either survival or NBD-PE accumulation were not evident with the nonphospholipogenic compounds, including compound D which consists of the right hand side aniline portion of the 5HT1_b_ antagonist compound A and which does not contain a cationic phenylpiperazine moiety.

### 3.2. Effect of Lysosomal Modulators on Survival Levels in Rat and Dog Hepatocytes Treated with Phospholipogenic Compounds

**Figure 4:**
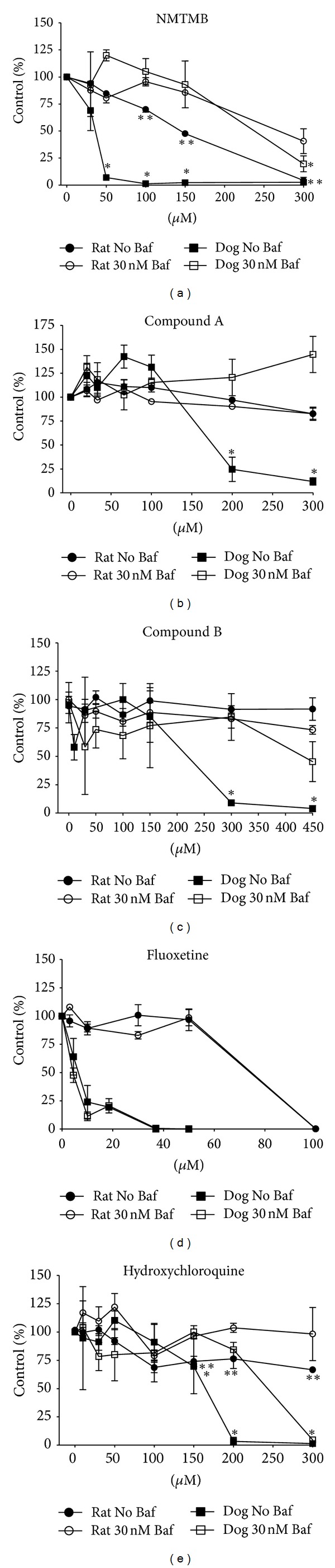
Effects of lysosomal modulator Baf (30 nM) cotreatments on 24 h survival of phospholipogenic-treated rat and dog hepatocytes. Cell survival was measured using the Cellomics Arrayscan (see Section 2). The data are expressed as an average of 3 replicates (±SD) for a single cell isolate. Statistically significant (*P* < 0.05, *t*-test) differences between dog phospholipogenic controls and modulator treatments occurred at the four top concentrations for NMTMB, top three concentrations for hydroxychloroquine, and top 2 concentrations for compounds A and B, and are illustrated by “ *”. For rat, statistically significant differences between phospholipogenic control and modulator treatments occurred at the top 3 concentrations for NMTMB and hydroxychloroquine and are illustrated by “ **”.

Lysosomes maintain their low pH via the proton transport activity of vacuolar ATPases. Baf is a macrolide antibiotic that acts as an inhibitor of vacuolar ATPases and therefore reduces lysosomal acidity. Baf was shown to prevent accumulation of the phospholipogenic compound hydroxychloroquine in Hela cells and reduced apoptosis caused by hydroxychloroquine treatment [18]. To address the question of whether phospholipogenic compounds are cytotoxic to hepatocytes by mechanisms involving lysosomes, effects of Baf on rat and dog hepatocytes were assessed in the presence of phospholipogenics. Results of experiments are shown in Figure 4. Dog hepatocyte survival in response to four of five phospholipogenics was significantly improved by cotreatment with 30 nM Baf. Rescue was conferred specifically against treatments of compounds from the same phenylpiperazine-containing AZ chemical series as well as hydroxychloroquine, but not for the more potent phospholipogenic fluoxetine in a singular experiment (*N* = 1) (or potent phospholipogenics amiodarone and perhexiline (*N* = 1), data not shown). In contrast to dog conditions, Baf cotreatment improved rat hepatocyte survival only against hydroxychloroquine (marginally and may not be biologically significant since hydroxychloroquine was not very cytotoxic in the rat control condition (~25–35% change in survival)) and NMTMB.

NMTMB was used subsequently for mechanistic investigations in rat cells as a representative tool to further probe common mechanisms mediated by the phenylpiperazine-containing AZ series because the phospholipidotic responses it caused in both species preparations were robust, and because cells treated with this compound were rescuable by Baf.

To confirm whether rescue is conferred by lysosomal modulation, rat hepatocytes treated with 300 *μ*M NMTMB were cotreated with a second lysosomal modulator, PADK, that operates differently than Baf. Butler et al. [20] showed PADK treatment upregulated active intralysosomal cathepsin D in hippocampal slices and to protect against chloroquine-mediated cytoxicity. In our experiment, PADK caused statistically significant rescue (average ± std dev % survival): 51 ± 8.5 for NMTMB plus PADK versus 9.3 ± 4% for NMTMB alone (*P* < 0.05, *t*-test).

### 3.3. Effects of NMTMB Mitochondrial Polarization in Rat Hepatocytes

**Figure 5:**
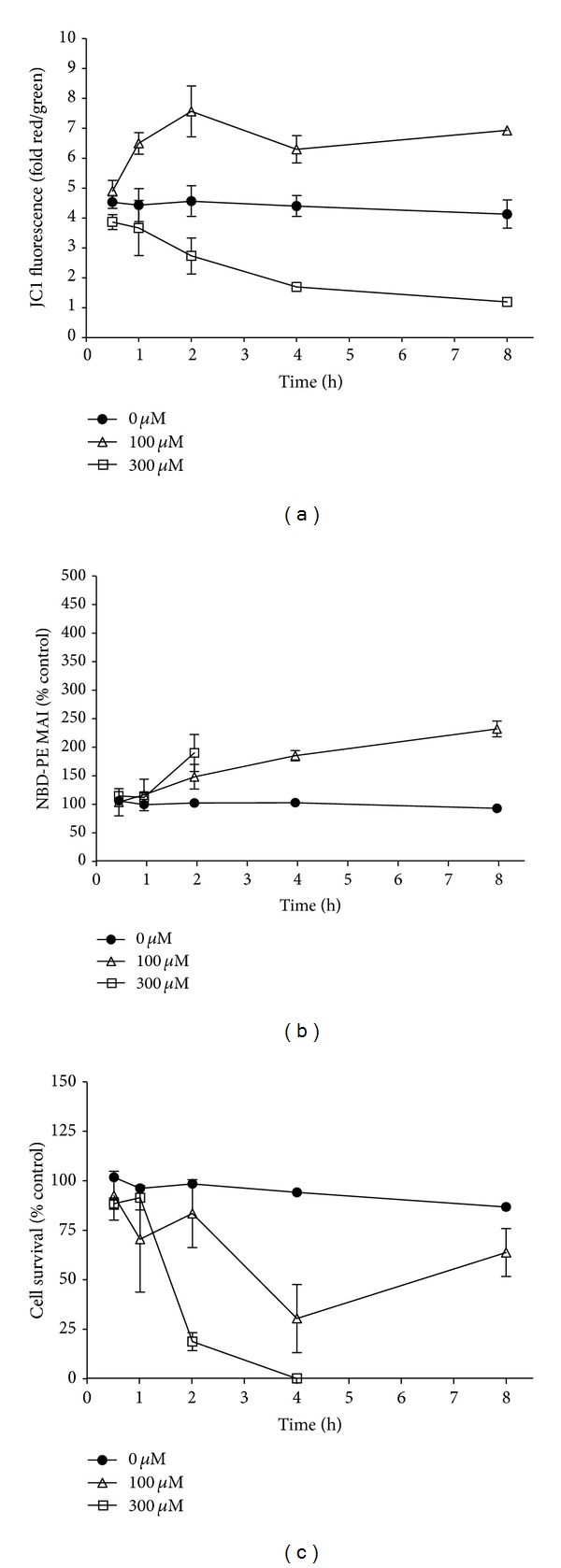
Effect of NMTMB on cell survival, NBD-PE accumulation, and mitochondrial polarization (JC-1 fluorescence) over 8 hours. Rat hepatocytes were treated with NMTMB for 30 minutes, 1 h, 2 h, 4 h, or 8 h. The data are expressed as an average of 3 (±SD) replicates from a single cell isolate. A statistically significant difference in JC-1 and NBD-PE levels between corresponding control replicates and that for NMTMB conditions was achieved by 1 h and 2 h, respectively (*P* < 0.05, *t*-test). As there was minimal survival observed for the higher test concentration beyond 2 h, NBDPE levels are not presented beyond 2 h.

The work of Boya et al. [18] demonstrated that hydroxychloroquine-mediated Hela cell mitochondrial membrane permeabilization occurred secondary to lysosomal destabilization. Similarly, we sought to determine whether mitochondrial or lysosomal mechanisms were mediating phospholipogenic-induced hepatocyte cytotoxicity.  Figure 5 shows an 8 hr time-course of mitochondrial polarization status (as indicated by JC-1 fluorescence measurement), NBD-PE accumulation and rat cell survival following NMTMB treatments at concentrations that bracketed the 24 h survival IC_50_ (159 *μ*M) and approached the approximate NBD-PE EC_50_ (half maximal effective concentration, ~65 *μ*M). At 100 *μ*M NMTMB, a mild but statistically significant (*P* < 0.05, *t*-test) mitochondria hyperpolarization occurred within 1 h of treatment, *before* NBD-PE accumulation was evident. Hyperpolarization increased over time, reaching a maximum by 2 h. Cell survival during this time period variably dropped by 40%, that is, sixty % of the cells survived the 8 h treatment. At 300 *μ*M NMTMB statistically significant (*P* < 0.05) mitochondria depolarization was observed within the first hour and this preceded detectable NBD-PE signal elevation as well. By 2 h, there was a statistically significant (*P* < 0.05) increased NBD-PE accumulation and significant cell death (20% survival compared to control), and by 4 h cell death was complete.

Similarly, for other phospholipogenics employed, when using concentrations that bracketed both 24 h survival IC_50_ (half maximal inhibitory concentration) values and approximated NBD-PE EC_50_ (half maximal effective concentration) values, hyperpolarization occurred at the lower test concentrations and depolarization was evident only when the 24 h IC_50_ values were exceeded (data not shown). Treating rat cells with phospholipogenics having 24 h survival IC_50_ values >300 *μ*M yielded only hyperpolarization. In all cases, mitochondrial membrane polarity changes were evident by 30 min to 1 h and small NBD-PE increases were evident by 2 hr. Thus, for every phospholipogenic compound test condition, the mitochondrial changes occurred approximately 1 h or earlier than detectable phospholipidotic responses.

### 3.4. Baf Modulation of Mitochondrial Polarization State in NMTMB-Treated Rat Hepatocytes

**Figure 6:**
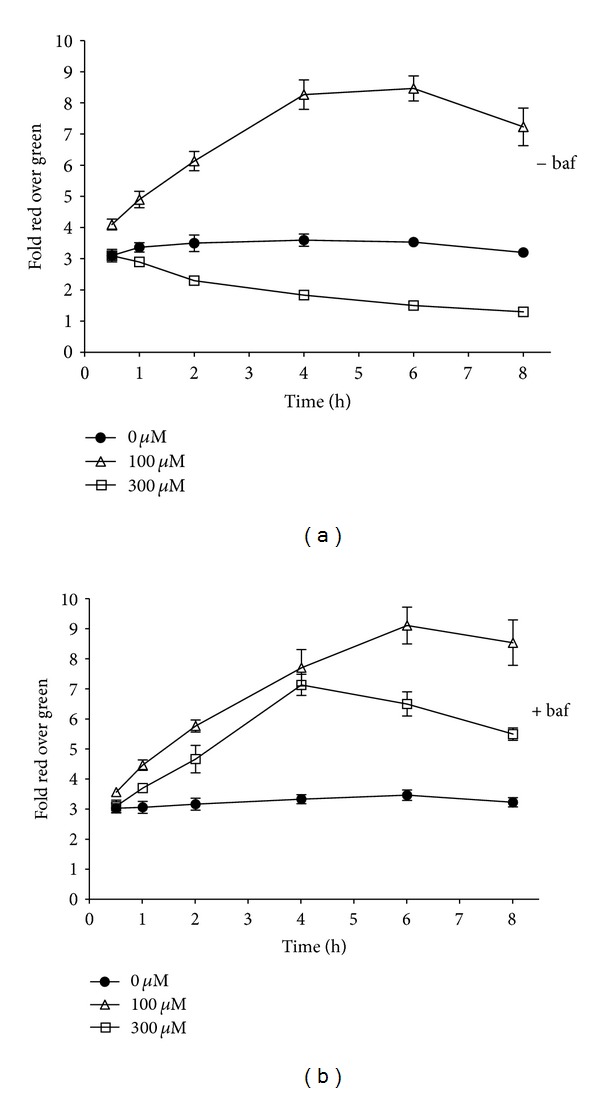
Baf (30 nM) cotreatment shifted the mitochondrial polarization state of rat hepatocytes treated with NMTMB over an 8-hour time course. Rat hepatocytes were treated with NMTMB for 30 minutes, 1 h, 2 h, 4 h, 6 h or 8 h. Mitochondrial polarization status was monitored using JC-1 fluorescence on a Molecular Devices Flexstation instrument. The data are expressed as an average of 3 replicates (±SD) from a single cell isolate. Statistically significant differences between control replicates and that for all NMTMB conditions was achieved by 1 h (*P* < 0.05, *t*-test).

To test whether lysosomal modulation affects mitochondrial membrane polarization, rat hepatocytes were cotreated with NMTMB and Baf, and JC-1 fluorescence was monitored over an 8 hr time-course (Figure 6). By 1 h Baf caused a marked shift from mitochondrial depolarization to a hyperpolarized state at the higher, 300 *μ*M NMTMB test concentration. Consistent with Baf cotreatment 24 h rescue shown in Figure 4, mitochondria from Baf cotreated cells never reached a depolarized state by 8 h.

### 3.5. Time-Course of AO Granular Staining Loss and Cell Death in NMTMB-Treated Rat Hepatocytes

**Figure 7:**
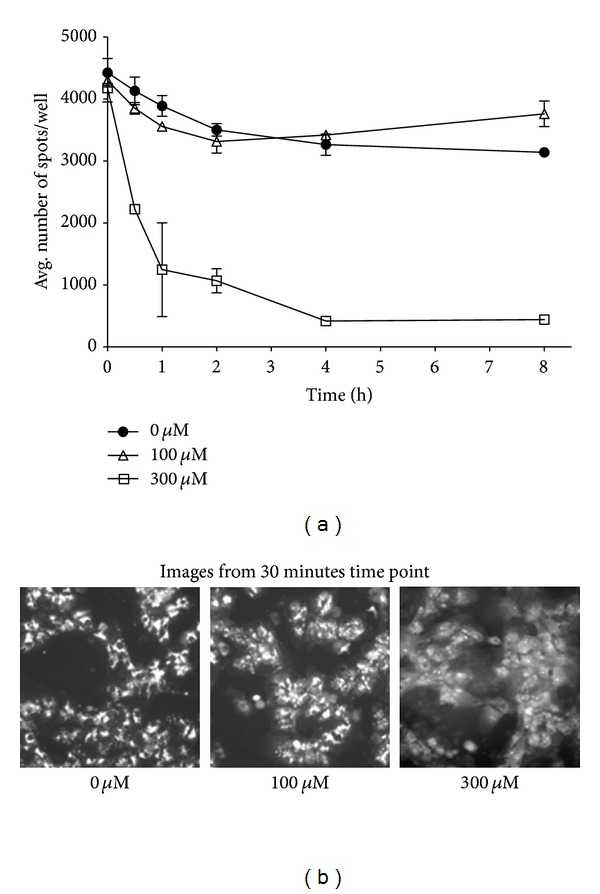
NMTMB affected acridine orange (AO) staining of lysosomes of rat hepatocytes over an 8-hour time course. AO was added to hepatocyte media for 10 minutes, removed, and replaced with media containing compound. Treatment with NMTMB proceeded for 30 minutes, 1 h, 2 h, 4 h or 8 h. AO staining was monitored using the Cellomics Arrayscan instrument. The data are expressed as an average of 3 replicates (±SD) from a single cell isolate. A statistically significant difference between control replicates and that for the NMTMB high test concentration is achieved by 30 min (*P* < 0.05, *t*-test) where marked cytotoxicity is evident by 4 hr (see Figure 5). Images from the Cellomics at the 30-minute time point are presented in (b).

The work by Boya et al. [18] suggested that hydroxychloroquine caused Baf-inhibitable lysosomal membrane destabilization, leading to cathepsin release and cell death. Since mitochondrial changes occurred prior to measurable phospholipidotic responses (elevated NBD-PE) in our experiments with NMTMB, we tested whether lysosomal membrane integrity was intact at early time points. To do this, we measured lysosomal staining with AO, a fluorescent dye that partitions into lysosomes. The fluorescent images in Figure 7 show robust/distinct, perinuclear, punctate staining of control cells and in cells from the lower concentration treatment throughout the time-course. In contrast, the higher, more cytotoxic, test concentration exhibited a combination of both diffuse cellular and punctuate AO fluorescence by 30 min. The spot count for the 100 *μ*M condition maintained or exceeded control levels of AO stained spots for up to 8 h. Within 30 minutes of treatment, the AO spot count in 300 *μ*M treated rat hepatocytes declined nearly 50% compared to control, and continued to drop to near 0% by 4 h. These early changes correlate with measures of cell death by 4 h (Figure 5) and suggest an early loss of lysosomal membrane integrity at 300 but not 100 *μ*M treatment. These effects occur concomitantly with mitochondrial membrane depolarization, but not hyperpolarization, and apparently prior to NBD-PE signal elevation.

## 4. Discussion

In this study dog and rat hepatocytes were treated with a variety of phospholipogenics to identify the more sensitive species and to test whether lysosomal or mitochondrial changes were the primary cause of cytotoxicity. We report that, against a panel of phospholipogenic treatments, dog hepatocytes exhibited lower 24 h survival IC_50_ (half maximal inhibitory concentration) values and generally exhibited a diminished capacity to accumulate NBD-PE, that is, dog hepatocytes appeared less able to process phospholipogenics and mount phospholipidotic protective responses across broad concentrations ranges. These differences appeared specific to phospholipogenic chemistries as they were not apparent following treatment with a limited test set of nonphospholipogenic (negative control) compounds, including compound D which consists of the right hand side aniline portion of the 5HT1_b_ antagonist compound A and which does not contain a cationic phenylpiperazine moiety. Indeed, for negative control treatment conditions, NBD-PE did not accumulate significantly, dog and rat hepatocytes died at similar test concentrations, and cells treated with these agents were unresponsive to Baf cotreatment.

Rat hepatocyte data indicate that both lysosomal destabilization and mitochondrial toxicity played key roles in phospholipogenic-mediated cytotoxicity and that phospholipid accumulation does not. NMTMB-induced loss of AO punctate fluorescence and the appearance of diffuse AO fluorescence alongside mitochondrial membrane depolarization. Second, Baf, a macrolide antibiotic that acts as an inhibitor of vacuolar ATPases and reduces lysosomal acidity and drug accumulation, shifted rat mitochondria to a hyperpolarized state from a depolarized state that is more clearly associated with acute cytotoxicity. Third, an additional lysosomal modulator (PADK) known to cause upregulation of intralysosomal and protective enzymes, including cathepsins B, D and L [20, 21], also prevented rat cytotoxicity caused by NMTMB. (This proposed mechanism of protection does not contradict, and should not be confused with, a cathepsin proapoptotic property once cathepsins are released from the lysosome under membrane destabilizing conditions.)

Mechanisms of hydroxychloroquine-mediated cell death have also been hypothesized by Boya et al. [18] to include the lysosome. Hydroxychloroquine-induced lysosomal membrane permeabilization led to pivotal Hela cell release of the acid protease cathepsin B from lysosomes, which in turn caused mitochondrial membrane permeabilization, release of cytochrome c, and activation of the caspase 3-mediated apoptotic pathway. Under these circumstances cathepsin release is proapoptotic. Cotreatment of the cells with the lysosomal modulator Baf prevented these changes as well as accumulation of hydroxychloroquine in lysosomes. Finally, Baf-mediated rescue for other cell contexts was reported by Shacka et al [4]. They showed protection against chloroquine-induced, caspase 3-driven apoptosis of cerebellar granule cells.

When cotreated with lysosomal modulators known to confer protective biochemical responses, dog hepatocytes were generally more rescuable than rat hepatocytes from phospholipogenic-induced cytotoxicity, including that for the AZ phenylpiperizine series. Rescue conferred by Baf was observed in rat cells only when treated with hydroxychloroquine (marginally, as hydroxychloroquine was not very cytotoxic in the rat control condition) or NMTMB, the later being the most cytotoxic in the AZ phenylpiperazine series to both species preparations. As the weaker phospholopogenic AZ compounds were DMSO solubility-limited, it was not possible to determine whether Baf would prevent rat cell death at higher test concentrations of these compounds. The data otherwise suggest that this subset of compounds caused distinct mechanisms of cytotoxicity involving both lysosome and mitochondria, which can be abrogated by cotreatment with lysosomal modulation, and whose phenotypic responses were not fully shared by Fluoxetine.

Theoretically, if dog lysosomes have a diminished capacity to sequester phospholipogenics into lysosomes for cellular protection (so-called depot effect), they may accumulate high concentrations in the cytoplasm, be free for electrophoretic attraction to the mitochondrial inner membranous space and cause membrane polarization and subsequent ATP depletion. The greater lysosomal partitioning of NBD-PE in response to phospholipogenics in rat relative to dog hepatocytes suggests that the rat hepatocyte basal functional capacity may be larger. We do not know, however, whether these differences are driven by differences in morphology, levels or type of phospholipid substrates, or acidity. We did not observe differences between dog and rat hepatocyte control levels of the lysosomal markers Lysotracker, AO, or NBD-PE in short-term cultures (data not shown).

An important caveat on differences in dog versus rat cytotoxicity responses is that cytotoxicity could be a function of reactive metabolite generation. We did not profile microsomal capacity or metabolite generation from either dog or rat preparations and assume that cytotoxicity is driven primarily by parent molecules.

Baf exhibits additional biological activities, as exemplified by the recent work of Teplova et al. [22]. Using isolated rat mitochondria, they demonstrated that Baf is a mitochondrial potassium ionophore. It caused uptake of potassium by energized mitochondria, mitochondrial swelling, loss of membrane potential, uncoupling of oxidative phosphorylation, and inhibition of maximal respiration rates. The minimal concentration required to affect these changes were 50 nM, a level that falls in the effective range used for our whole cell experiments. However, since we do not know the intracellular Baf levels in our experiment, it is not possible to make a direct comparison to the data of Teplova et al. [22]. Furthermore, the effects on mitochondria described by them do not elucidate a mechanism of rescue for phospholipogenic treatment cell contexts in our study as they represent cytotoxic attributes, not protective ones.

In summary, our data add to the body of literature describing a complex picture of differences in phospholipogenic *in vitro* cytotoxicity relating to structure, phospholipogenic potency, and multiplicity of cellular effects in two species. We demonstrated that dog hepatocytes were with select compounds less capable of mounting an adaptive, protective phospholipidotic response than were rat hepatocytes, and both lysosomal and mitochondrial changes appear to be linked mechanistically to phospholipogenic-mediated cytotoxicity *in vitro*. It would be of interest to confirm in a comprehensive survey of *in vivo* toxicology studies whether the *in vitro* response profiles described here correlate with *in vivo* liver PLD and liver toxicity responses as they do for compound A. In addition, a comparison to hepatocytes from preclinical species to human is warranted in order to shed light on prediction/translatability of these cytotoxicity profiles.

## Figures and Tables

**Table 1:** Cell survival IC_50_ (half maximal inhibitory concentration) values of dog and rat hepatocytes treated with phospholipogenic or nonphospholipogenic compounds. Cells were fixed with formaldehyde/Hoescht, and survival was quantified using the Cellomics Arrayscan instrument (see Materials and Methods). Isolates were obtained on different days. The data are represented as individual experiments only (*N* = 1 or 2) or as an average of 3 individual (separate isolates, *N* = 3) experiments ± SD.

	Dog	Rat
	Survival IC_50_ *μ*M	Survival IC_50_ *μ*M
Phospholipogenic compounds		

NMTMB	37 ± 13^a^	148 ± 41
A	298 ± 22	>450 (3)
B	153 ± 63	>300 (3)
Hydroxychloroquine	90, 159	220, >300
Fluoxetine	19	61

Nonphospholipogenic compounds		

Diclofenac	656	832
C	>300	>300
D	>300	>300

^a^Statistically significant (*P* < 0.05, *t*-test) differences observed in species' response.

## Abbreviations

AO: Acridine orange
Baf: Bafilomycin A1
DMSO: Dimethyl sulfoxide
NBD-PE: N-(7-nitrobenz-2-oxa-1,3-diazol-4-yl)-1,2-dihexadecanoyl-snglycero-3-phosphoethanolamine, triethylammonium salt
PLD: Phospholipidosis
NMTMB: N-[(2R)-5-methyl-8-(4-methylpiperazin-1-yl)tetralin-2-yl]-4-morpholino-benzamide.
